# miR‐4732‐5p promotes breast cancer progression by targeting TSPAN13

**DOI:** 10.1111/jcmm.14145

**Published:** 2019-01-31

**Authors:** Ya‐Wen Wang, Song Zhao, Xun‐Yi Yuan, Yao Liu, Kai Zhang, Jianli Wang, Jiang Zhu, Rong Ma

**Affiliations:** ^1^ Department of Breast Surgery Qilu Hospital of Shandong University Jinan Shandong People's Republic of China; ^2^ Department of Physiology and Pathophysiology School of Basic Medicine Shandong University Jinan Shandong People's Republic of China

**Keywords:** breast cancer, metastasis, miR‐4732‐5p, proliferation, TSPAN13

## Abstract

MiR‐4732‐5p was previously found to be dysregulated in nipple discharge of breast cancer. However, the expression and function of miR‐4732‐5p in breast cancer remain largely unknown. Here, the expression of miR‐4732‐5p was detected using quantitative real‐time PCR in breast cancer tissues and cell lines. Cell proliferation, apoptosis, migration and invasion assays were performed to examine the effects of miR‐4732‐5p in breast cancer. In addition, mRNA sequencing, bioinformatics analysis, Western blot and luciferase assays were performed to identify the target of miR‐4732‐5p. Overall, miR‐4732‐5p was significantly down‐regulated in breast cancer tissues, especially in lymph node metastasis (LNM)‐negative tissues, compared with adjacent normal tissues. However, it was more highly expressed in LNM‐positive breast cancer tissues, compared with LNM‐negative ones. Expression of miR‐4732‐5p was positively correlated with lymph node metastasis, larger tumour size, advanced clinical stage, high Ki‐67 levels and poor prognosis. MiR‐4732‐5p promoted cell proliferation, migration and invasion in breast cancer. MiR‐4732‐5p directly targeted the 3′‐UTR of tetraspanin 13 (TSPAN13) and suppressed TSPAN13 expression at the mRNA and protein levels. These results suggested that miR‐4732‐5p may serve as a tumour suppressor in the initiation of breast cancer, but as a tumour promoter in breast cancer progression by targeting TSPAN13.

## 1 INTRODUCTION

Breast cancer is the most frequently diagnosed cancer and the leading cause of cancer death in women worldwide.^1^ It is not the primary tumour, but its metastases are the main reason of death.^2^ The precise molecular circuitry that governs the metastasis process has not been completely understood. It is urgent to identify novel biomarkers and therapeutic targets to predict and mitigate metastases in breast cancer patients.

MicroRNAs (miRNAs, miRs) are small non‐coding RNAs (~22 nucleotides) that regulate gene expression at the post‐transcriptional level.^3^ MiRNAs have been shown to play critical roles in many physiological and pathological processes.^3^, ^4^, ^5^, ^6^ Furthermore, miRNAs are involved in cancer initiation and progression, consequently regulating the processes of invasion and metastasis in several types of cancer.^7^, ^8^ MiR‐10b and miR‐335 were the first miRNAs to be reported as promoters or inhibitors of metastasis respectively.^9^ In breast cancer, certain miRNAs have been associated with the invasion‐metastasis cascade, including miR‐10b, miR‐21, miR‐31, miR‐373 and miR‐520c.^10^ MiR‐4732‐5p is a novel miRNA that has not been well studied in human cancers. In our previous study, miR‐4732‐5p was found to be down‐regulated in breast cancerous nipple discharge compared with benign nipple discharge, supporting that it might serve as a potential tumour biomarker for breast cancer detection.^11^ However, the expression and function of miR‐4732‐5p remain largely unknown.

Here, we provide evidence that miR‐4732‐5p was down‐regulated in lymph node metastasis (LNM)‐negative breast cancer tissues; however, it was overexpressed in LNM‐positive tissues. MiR‐4732‐5p was positively associated with aggressive clinical parameters, poor prognosis and cell biological behaviours. Tetraspanin 13 (TSPAN13) was a direct target of miR‐4732‐5p.

## 2 MATERIALS AND METHODS

### 2.1 Tissue samples

A total of 67 pairs of primary breast cancers and corresponding normal tissues were obtained from breast cancer patients at the Qilu Hospital of Shandong University (Jinan, China) from 2015 to 2017. The fresh specimens were immediately stored in liquid nitrogen until RNA was extracted. The patients’ clinicopathological characteristics are summarized in Table 1. Written informed consent was obtained from each patient. The study was approved by the Ethics Committee of Qilu Hospital of Shandong University, and was carried out according to the World Medical Association Declaration of Helsinki.

**Table 1:** Clinical details of breast cancer patients

Parameters	Number
Age (Median with range)	51 (26‐84)
pT stage (tumour size)	
pTis	3
pT1 (≤2cm)	28
pT2 (2<T≤5)	33
pT3 (>5)	3
Lymph node metastasis (N stage)	
pN0 (0)	30
pN1 (1‐3)	14
pN2 (4‐9)	16
pN3 (≥10)	7
Oestrogen receptor (ER) status	
Negative	11
Positive	56
Progesterone receptor (PR) status	
Negative	13
Positive	54
HER2 status	
Negative	46
Positive	13
Unknown	8
Ki‐67	
Negative (≤14%)	17
Positive (>14%)	50
Clinical TNM stage	
0	3
I	16
II	24
III	24

### 2.2 Cell culture

Human breast cancer lines (MCF‐7, T47D, SK‐BR‐3, ZR‐75‐1, MDA‐MB‐453, BT549, MDA‐MB‐468, MDA‐MB‐231 and MDA‐MB‐157) and the non‐tumourigenic cell line MCF10A were obtained from Cell Resource Center, Institute of Basic Medical Sciences, Chinese Academy of Medical Sciences (Beijing, China) and cultured under standard conditions.

### 2.3 Cell transfection

The miR‐4732‐5p mimics and negative control were obtained from GenePharma (Shanghai, China). MiRNA mimics are small, chemically modified, double‐stranded RNA molecules that mimic endogenous mature miRNA molecules and widely used for short‐term gain‐of‐function miRNA experiments.^12^ MiRNA mimics and negative control transfection was carried out with the X‐tremeGENE transfection reagent (Roche) according to the manufacturer's instructions. For stable expression of miR‐4732‐5p, cells were transfected with lentivirus miR‐4732‐5p‐expressing vector LV3‐hsa‐miR‐4732‐5p or a negative control LV3NC (GenePharma, Shanghai, China), which expressed GFP (green fluorescent protein) as a marker. The expression of GFP was monitored under a fluorescence microscope. The transfected cells were screened with puromycin for the ones stably expressing miR‐4732‐5p.

### 2.4 Cell proliferation assay

The proliferation ability of cells was monitored using the MTS 3‐(4, 5‐dimethylthiazol‐2‐yl)‐5‐(3‐carboxymethoxyphenyl)‐2‐(4‐sulfophenyl)‐2H‐tetrazolium assay (CellTiter 96^®^ AQueous One Solution Cell Proliferation Assay; Promega, Madison, WI, USA), as previously described.^13^

### 2.5 Colony formation assay

Cells (300‐500 per well) were seeded in six‐well plates. After incubation for 2 weeks, colonies were fixed and stained.

### 2.6 Migration and invasion assay

Cell migration capability was detected by using Transwell chambers (Corning, NY, USA) as previously described.^4^ Transwell chambers pre‐coated with the Matrigel matrix (BD Biosciences) were used for cell invasion assay.

### 2.7 RNA extraction and quantitative real‐time PCR (qRT‐PCR)

Total RNA was extracted with Trizol reagent (Invitrogen, Carlsbad, CA, USA). cDNA synthesis and quantitative real‐time PCR (qRT‐PCR) were performed with the All‐in‐One^™^ miRNA qRT‐PCR Reagent Kit (GeneCopoeia) using a CFX^™^ 96 C1000 Real‐Time machine, with the RNU6B small nuclear RNA as internal normalized reference. The miR‐4732‐5p and RNU6B primers were obtained from GeneCopoeia.

### 2.8 mRNA sequencing

Total RNA from miR‐4732‐5p or negative control‐transfected cells (n = 3 for each) was extracted using Trizol reagent (Invitrogen) and was subsequently used for removing the rRNAs using Ribo‐Zero rRNA Removal Kits (Illumina, San Diego, CA, USA). RNA libraries were constructed using rRNA‐depleted RNAs with TruSeq Stranded Total RNA Library Prep Kit (Illumina). Libraries were controlled for quality and quantified using the BioAnalyzer 2100 system (Agilent Technologies, Inc., USA). 10 pM libraries were denatured as single‐stranded DNA molecules, captured on Illumina flow cells, amplified in situ as clusters and finally sequenced for 150 cycles on Illumina HiSeq Sequencer. Paired‐end reads were harvested from Illumina HiSeq 4000 sequencer, and were quality controlled by Q30. After 3′ adaptor‐trimming and low quality reads removing by cutadapt software (v1.9.3), the high quality clean reads were aligned to the reference genome (UCSC hg19) with hisat2 software (v2.0.4). Then, guided by the Ensembl gtf gene annotation file, cuffdiff software (part of cufflinks) was used to get the gene level FPKM as the expression profiles of mRNA, and fold change (FC) and *P* value were calculated based on FPKM, differentially expressed mRNA (FC > 2, *P* < 0.05) were identified.

### 2.9 Luciferase assay

The wild‐type (WT) TSPAN13 3′‐UTR fragments or the mutant (without the predicted miR‐4732‐5p target sequence), were inserted downstream of the firefly luciferase gene in the pmirGLO (Generay) plasmid. The reporter plasmid pmirGLO‐TSPAN13 WT/Mutation, along with miR‐4732‐5p mimics or negative control, were transfected into cells. After transfection for 48 hours, cell lysates were collected, and luciferase activities were measured by the Dual Luciferase Reporter System (Promega, WI, USA) using a Tecan Infinite M200 Pro instrument. The luminescence intensity of firefly luciferase was normalized to that of Renilla luciferase.

### 2.10 Western blot

Cells were harvested and protein extracts were obtained with lysis buffer. Equal amounts of protein were electrophoresed on 10% SDS‐PAGE gels, and then transferred to PVDF membrane. After blocking, the membranes were incubated with anti‐TSPAN13 (1:500, abs128170, absin) and anti‐β‐actin (1:1000, BA2305, Boster), followed by incubation with horseradish peroxidase‐conjugated secondary antibodies. Proteins were visualized by using the AlphaView software (Version: 3.2.2.0) on a FluorChem Q machine and analysed by the Multi Gauge V3.2 software.

### 2.11 Statistical analysis

Differences between two groups were analysed by Student's *t* test, and ANOVA was used to find differences among three or more groups. Two‐sided *P* < 0.05 was considered significant. The web‐tools miRpower,^14^ BioProfiling^15^, ^16^ and PrognoScan^17^ were utilized to determine the prognostic value of miR‐4732‐5p and TSPAN13, respectively, in breast cancer using publicly available data.

## 3 RESULTS

### 3.1 Expression of miR‐4732‐5p in breast cancer tissues and cell lines

**Figure 1:**
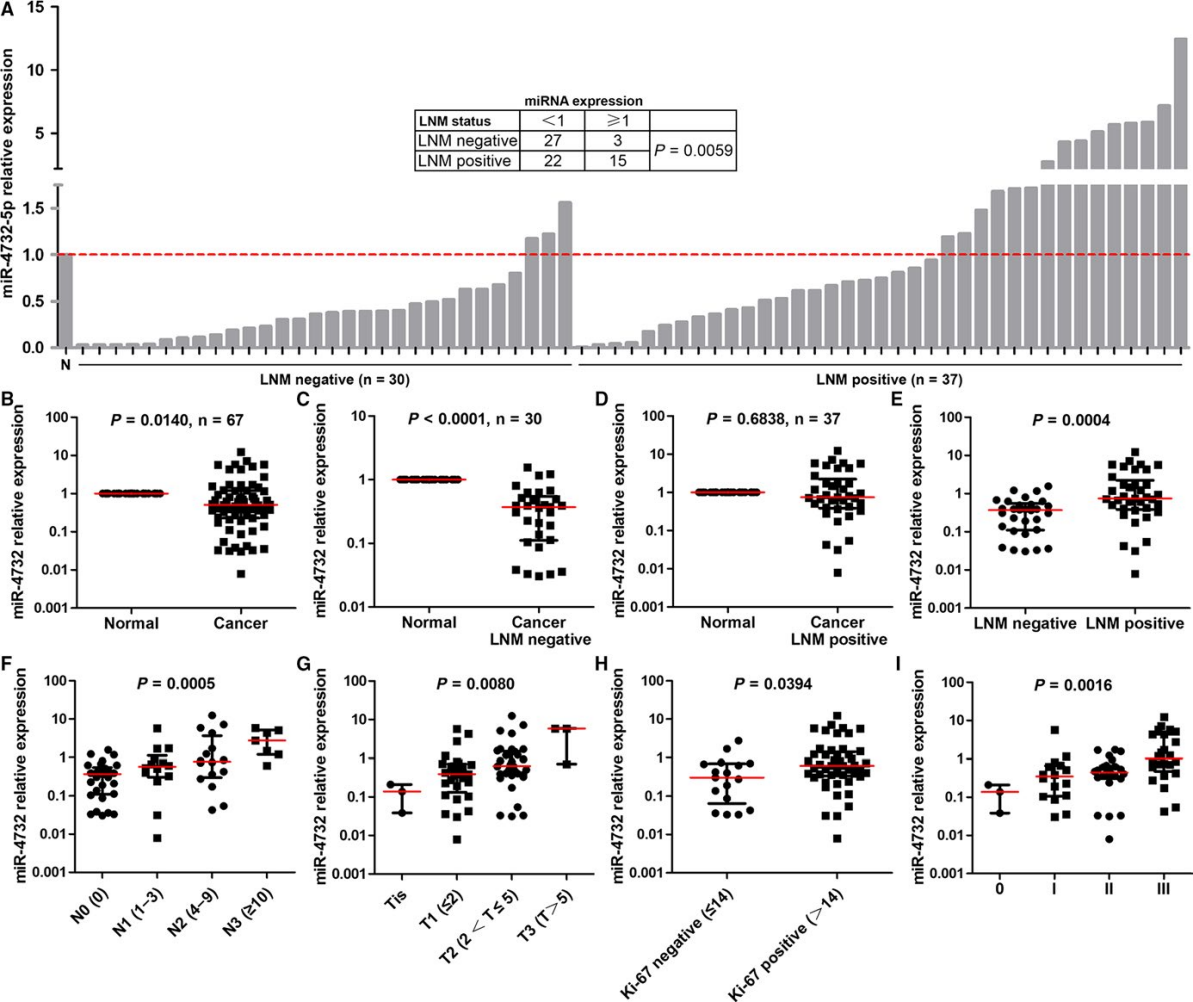
Expression of miR‐4732‐5p in breast cancer tissues and its association with clinicopathological parameters. (A‐E) Overall, miR‐4732‐5p was down‐regulated in breast cancer tissues, compared with the corresponding normal tissues, especially in lymph node metastasis (LNM)‐negative tissues (A‐C). However, LNM‐positive tissues displayed higher miR‐4732‐5p expression than lymph node metastasis (LNM)‐negative tissues (A, D, E). N, normal tissues. (F‐I) Expression of miR‐4732‐5p was positively correlated with lymph node metastasis (N stage, F), tumour size (T stage, G), Ki‐67 expression (H) and clinical stage (I)

**Figure 2:**
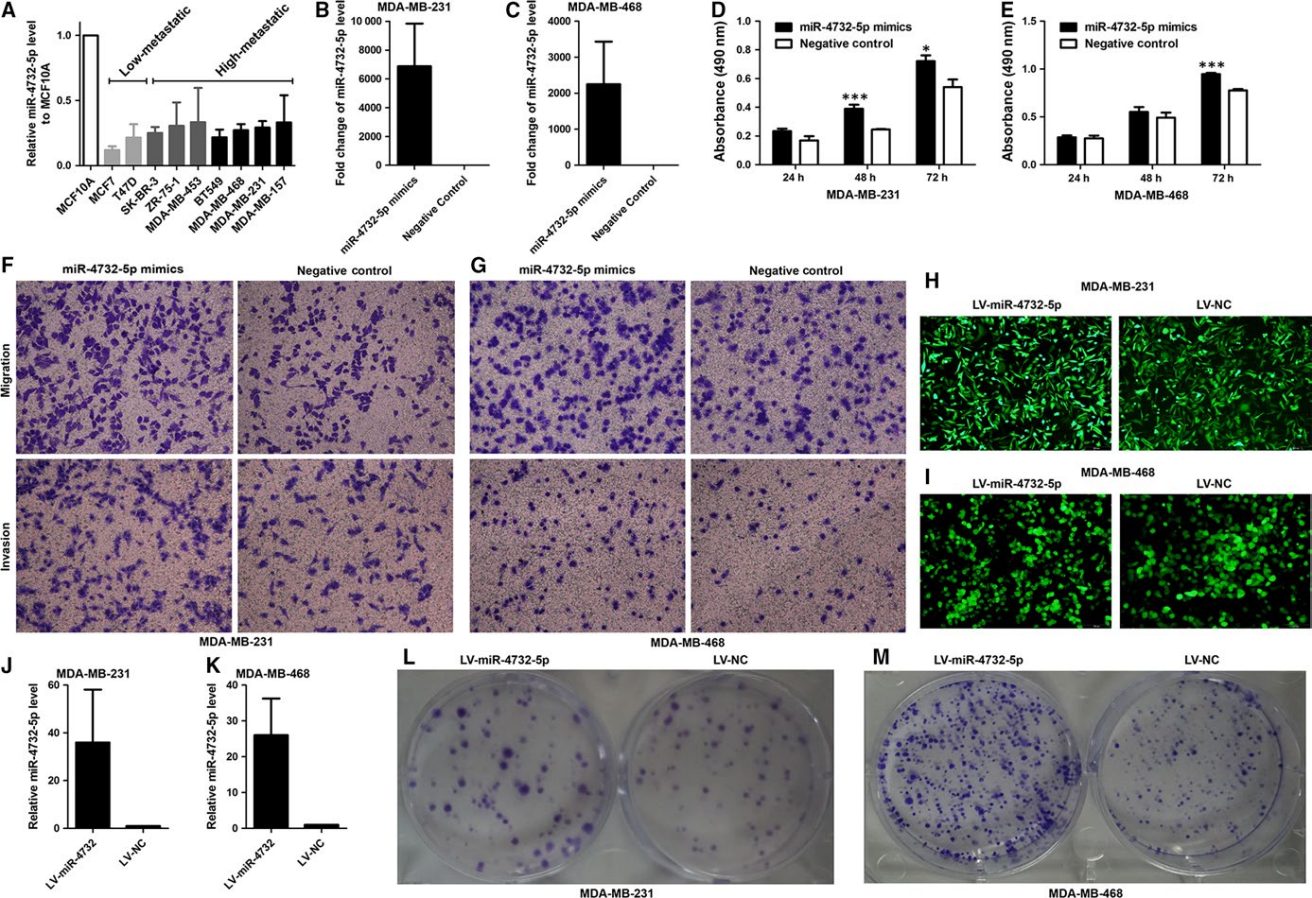
Expression of miR‐4732‐5p in breast cancer cell lines and its effect on cell biological behaviours. (A) miR‐4732‐5p was down‐regulated in breast cancer cell lines (n = 9) compared with the non‐tumourigenic cell line MCF10A. It is also noted that miR‐4732‐5p was relatively highly expressed in high‐metastatic cell lines than low‐metastatic cell lines. (B‐C) miR‐4732‐5p mimics transfection led to significant high expression of miR‐4732‐5p in breast cancer cells. (D‐E) Overexpression of miR‐4732‐5p promoted cell proliferation as revealed by MTS assays. (F‐G) MiR‐4732‐5p enhanced cell migration and invasion ability, compared with negative control. (H‐I) After lentivirus vector transfection, green fluorescence protein expression was observed by using fluorescence microscope. (J‐K) Lentivirus miR‐4732‐5p vector up‐regulated miR‐4732‐5p expression, compared with the control vector. (L‐M) Stable expression of miR‐4732‐5p expression increased colony formation in MDA‐MB‐231 and MDA‐MB‐468 cells. *P < 0.05; **P < 0.01

Expression of miR‐4732‐5p was detected in primary breast cancers and corresponding normal tissues. Among the 67 patients, 49 (73%) cases showed significant lower expression of miR‐4732‐5p in breast cancer tissues compared with normal tissues (*P* = 0.0140, paired *t* test, Figure 1A,B). Moreover, miR‐4732‐5p was found to be underexpressed in nine cancer cell lines compared to the non‐tumourigenic cell line MCF10A (Figure 2A).

### 3.2 Association between miR‐4732‐5p expression and clinicopathological parameters and prognosis

Lymph node metastasis (LNM) is one of the most important prognostic indicators for breast cancer and thus we are interested in the association between miR‐4732‐5p expression and LNM. According to the status of lymph node metastasis, we divided the cancer tissues into LNM‐negative and LNM‐positive groups. Interestingly, compared with normal breast tissues, miR‐4732‐5p was down‐regulated in LNM‐negative cancer tissues (Figure 1C, *P* < 0.0001), rather than LNM‐positive cancer tissues (Figure 1D, *P* = 0.6838). Specifically, 27/30 (90%) of the LNM‐negative cancer tissues expressed lower levels of miR‐4732‐5p; however, only 22/37 (41%) of the LNM‐positive cancer tissues displayed less miR‐4732‐5p level than normal breast tissues (Figure 1A, Fisher's exact test, *P* = 0.0059). Indeed, miR‐4732‐5p was significantly highly expressed in LNM‐positive cancers compared with LNM‐negative cancers (Figure 1E, *P* = 0.0004). Moreover, expression of miR‐4732‐5p increased along with N stage (lymph node metastasis) (Figure 1F, one‐way ANOVA, *P* = 0.0005). It is noted that high‐metastatic breast cancer cell lines (SK‐BR‐3, ZR‐75‐1, MDA‐MB‐453, BT549, MDA‐MB‐468, MDA‐MB‐231 and MDA‐MB‐157) expressed relatively higher levels of miR‐4732‐5p than low‐metastatic cell lines (MCF‐7 and T47D) (Figure 2A).

In addition, miR‐4732‐5p was found to be positively correlated with larger tumour size (Figure 1G, one‐way ANOVA, *P* = 0.0080), high Ki‐67 index (Figure 1H, *P* = 0.0394) and advanced clinical stage (Figure 1G, one‐way ANOVA, *P* = 0.0016).

As breast cancer is rather heterogeneous, the relationship between miR‐4732‐5p expression and subtypes of breast cancer was further investigated. Our data showed that miR‐4732‐5p expression showed no significant difference among the four molecular subtypes (Luminal A, Luminal B, HER2‐enriched and Triple negative) (Figure S1A, *P* > 0.05), or between ER+ and ER‐ (Figure S1B, *P* > 0.05), or PR+ and PR‐ (Figure S1C, *P* > 0.05), or HER2+ and HER2‐ (Figure S1D, *P* > 0.05) breast cancers. Furthermore, the breast cancer tissues were divided into different subgroups based on molecular subtypes (Luminal A, Luminal B, HER2‐enriched and Triple negative subgroups, Figure S2) or ER/PR/HER2 status (ER+, ER‐, PR+, PR‐, HER2+ and HER2‐ subgroups, Figure S3‐S5). Overall, our results showed that in each subgroup miR‐4732‐5p was down‐regulated in cancer tissues compared with normal tissues, and positively correlated with lymph node metastasis, larger tumour size and advanced clinical stage (Figure S2‐S5), although certain subgroup (eg triple negative cancer) included too small samples for analysis and several analyses did not reach statistical significance due to smaller sample size. We believe that the observations that miR‐4732‐5p was down‐regulated in breast cancer and correlated aggressive clinical feathers may be common in breast cancer, and not apply specifically to a specific subtype.

**Figure 3:**
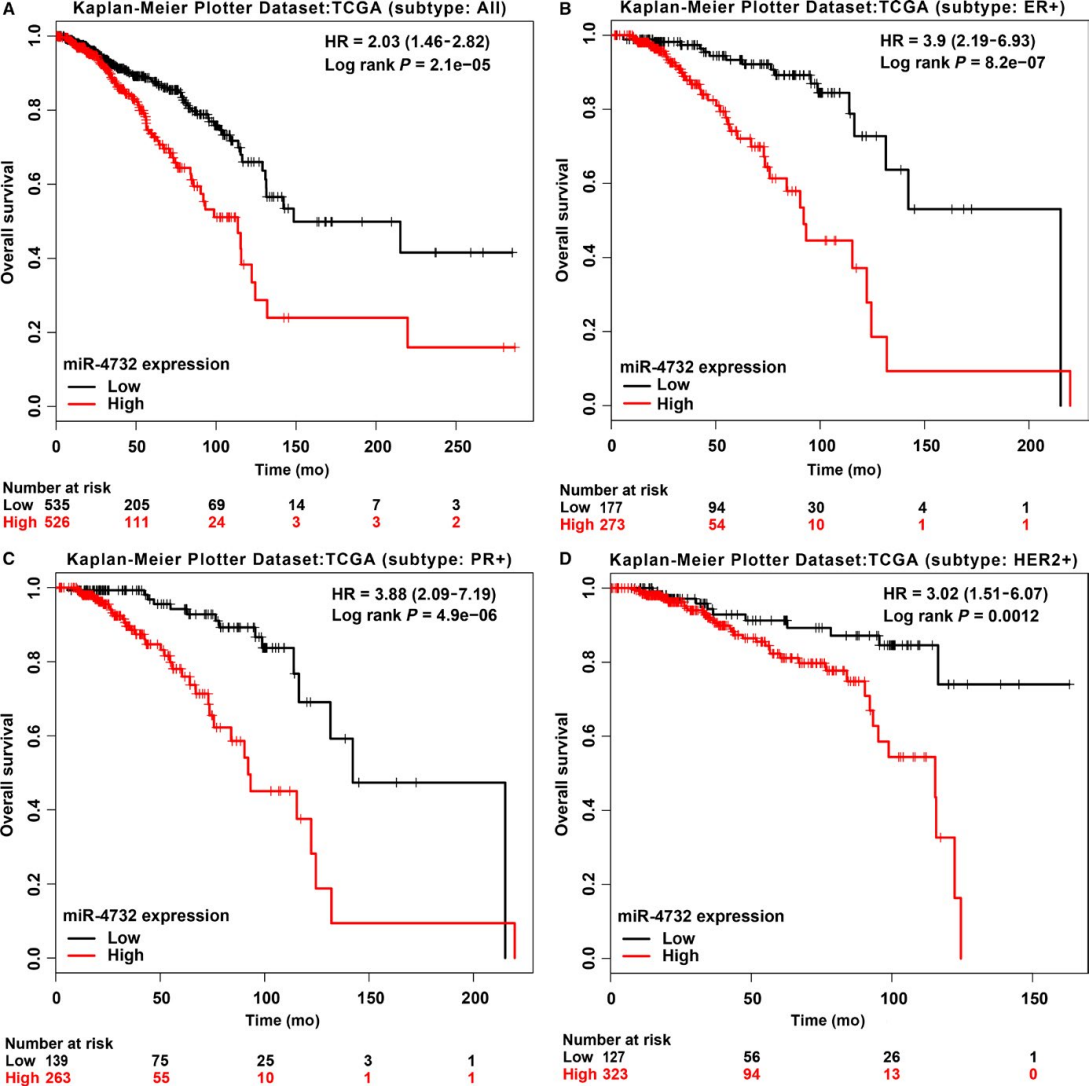
Prognostic value of miR‐4732‐5p in breast cancer based on the miRpower data. (A) Data from miRpower revealed that breast cancer patients with high expression of miR‐4732‐5p had poorer survival than those with low expression (total n = 1061, HR = 2.03 (1.46‐28.2), *P* = 2e‐5, TCGA dataset, median survival time (months): 113.63 vs. 148.53). (B‐D) Similar results were obtained in ER+, PR+, or HER2+ breast cancer patients

The prognostic role of miR‐4732‐5p was determined by the miRpower database.^14^ Our results showed that patients with high expression of miR‐4732‐5p (n = 526) had poorer survival than those with low expression of miR‐4732‐5p (n = 535, Figure 3A, HR = 2.03, *P* < 0.0001, TCGA dataset, all subtypes patients included). The median survival time of low expression group and high expression group was 148.53 and 113.63 months respectively. Interestingly, the miRpower database could also provide survival analysis in certain subtypes and we found that high expression of miR‐4732‐5p was also associated with worse prognosis in ER+, PR+ and HER2+ patients (Figure 3B‐D), suggesting that miR‐4732‐5p may be a risk prognostic factor independent of breast cancer subtypes.

**Figure 4:**
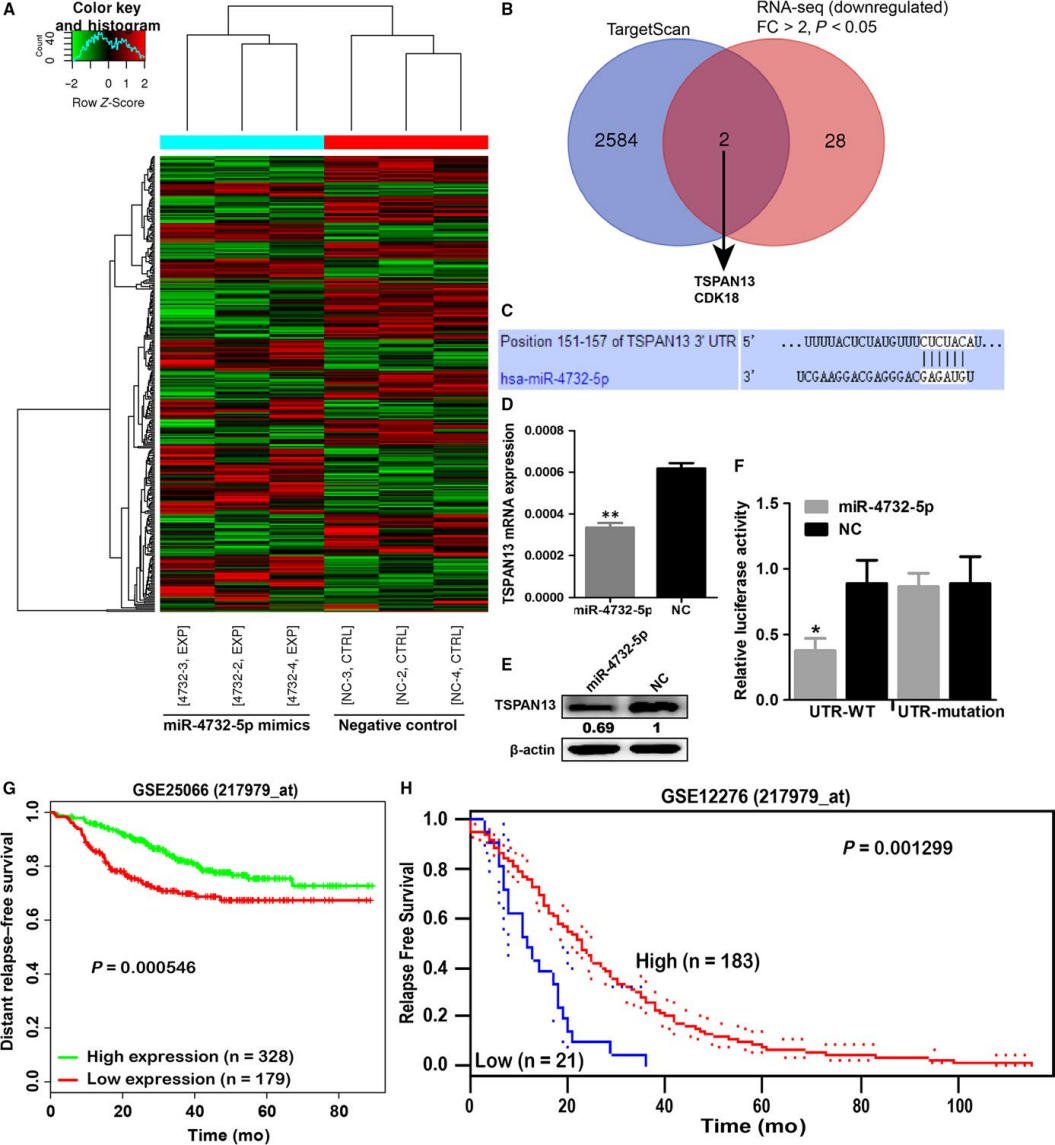
TSPAN13 was a direct target of miR‐4732‐5p and low expression of TSPAN13 was associated with poor prognosis of breast cancer patients. (A) MDA‐MB‐231 cells transfected with miR‐4732‐5p mimics (n = 3) or negative control (n = 3) were subjected to mRNA sequencing analysis to screen differentially expressed mRNAs. In total, 157 mRNAs were found to be up‐regulated, whereas 170 mRNAs were down‐regulated after miR‐4732‐5p mimics transfection (*P* < 0.05). Among them, 40 were up‐regulated and 30 were down‐regulated by more than twofold (fold change > 2, and *P* < 0.05) in the miR‐4732‐5p mimics group than the negative control group. (B) mRNA sequencing and TargetScan prediction indicated two potential target genes (TSPAN13 and CDK18) of miR‐4732‐5p. (C‐E) miR‐4732‐5p could down‐regulate mRNA and protein levels of TSPAN13. (F) TSPAN13 was a direct target of miR‐4732‐5p as revealed by luciferase assays. (G‐H) Data from BioProfiling (G) and PrognoScan (H) showed that low expression of TSPAN13 predicted poor survival of breast cancer patients. *P < 0.05; **P < 0.01

### 3.3 miR‐4732‐5p promoted proliferation, migration and invasion of breast cancer cells

To investigate the functional effects of miR‐4732‐5p, we performed gain‐of‐function studies using miRNA transfection of MDA‐MB‐231 and MDA‐MB‐468 cells, as these two cell lines displayed moderate expression of miR‐4732‐5p. qRT‐PCR analysis confirmed that miR‐4732‐5p mimics transfection could dramatically increase miR‐4732‐5p expression levels (Figure 2B‐C).

MTS proliferation assay demonstrated that cell proliferation was significantly enhanced by transfection of miR‐4732‐5p in comparison with the negative control transfectants (Figure 2D‐E). Cell migration activity was significantly increased by miR‐4732‐5p transfection of both MDA‐MB‐231 and MDA‐MB‐468 cell lines compared with negative control‐transfected cells (Figure 2F‐G). Moreover, in invasion assays, transfection with miR‐4732‐5p significantly promoted cell invasion compared with negative control‐transfected cells (Figure 2F‐G). However, miR‐4732‐5p demonstrated little effect on breast cancer cell apoptosis (Figure S6). To gain insight into the roles of miR‐4732‐5p in colony formation, miR‐4732‐5p was stably overexpressed in MDA‐MB‐231 and MDA‐MB‐468 cells by lentivirus vectors (Figure 2H‐K). Our data showed that miR‐4732‐5p increased colony formation capacity of breast cancer cells (Figure 2L‐M). These results indicated that miR‐4732‐5p could facilitate tumour progression by enhancing cell proliferation, migration and invasion.

### 3.4 Tetraspanin 13 (TSPAN13) was identified as a direct target of miR‐4732‐5p

To identify genes targeted by miR‐4732‐5p, we performed genome‐wide mRNA sequencing analysis using MDA‐MB‐231 cell line and selected down‐regulated genes (fold change > 2 and *P* < 0.05) by miR‐4732‐5p transfection compared with negative control transfection (Figure 4A). Among 30 putative candidate genes, two genes (TSPAN13 and CDK18) contained putative binding sites of miR‐4732‐5p in their 3′‐UTR regions, as revealed by the TargetScan database (Figure 4B). TSPAN13 was of particular interest in light of its reported roles in suppressing cancer cells progression.^18^, ^19^ qRT‐PCR and Western blot were performed to investigate whether miR‐4732‐5p altered TSPAN13 gene and protein expression. The mRNA and protein expression levels of TSPAN13 were significantly repressed in miR‐4732‐5p transfectants compared with negative control‐transfected cells (Figure 4C‐E).

Next, luciferase assay was performed to determine whether TSPAN13 mRNA carried a target site for miR‐4732‐5p. The TargetScan database predicted that one putative miR‐4732‐5p binding site existed in the 3′‐UTR of the gene (position 151–157). Data showed that the luminescence intensity was significantly reduced by co‐transfection with miR‐4732‐5p and the vector carrying the wild‐type 3′‐UTR of TSPAN13, rather than the mutant 3′‐UTR with deleted target sites (Figure 4F).

Furthermore, the prognostic value of TSPAN13 was determined using publicly available data. As shown in BioProfiling,^15^, ^16^ low expression of TSPAN13 predicted poor survival of breast cancer patients (GEO database: GSE250665, n = 507, *P* = 0.000546, Figure 4G). Results from the PrognoScan database^17^ showed that breast cancer patients with low expression of TSPAN13 had shorter survival time (GSE12276 dataset, n = 204, *P* = 0.001299, Figure 4H) than those with high TSPAN13 expression.

## 4 DISCUSSION

MiRNAs act as key regulators in cancer initiation and progression, including cell proliferation, apoptosis, differentiation, invasion, metastasis and drug resistance.^3^, ^4^, ^7^, ^10^, ^20^ MiRNAs play roles as either tumour suppressors or oncogenes by negatively regulating their target genes.^7^ For example, miR‐489 inhibits breast cancer proliferation, metastasis and chemoresistance by suppressing SPIN1‐mediated PI3K‐Akt pathway.^3^, ^21^ Ma et al shows that Twist‐induced expression of miR‐10b initiated tumour invasion and metastasis in breast cancer.^22^

In this study, miR‐4732‐5p was found to be down‐regulated in breast cancer tissues compared with adjacent normal tissues. However, increased expression of miR‐4732‐5p was found in cancer tissues with lymph node metastasis, larger tumour size, high Ki‐67 index and advanced clinical stage, independent of cancer subtypes. High expression of miR‐4732‐5p was associated with poor prognosis in breast cancer. MiR‐4732‐5p significantly promoted cell proliferation, colony formation, migration and invasion in breast cancer cells. Mechanistically, TSPAN13 was identified as a direct target of miR‐4732‐5p and low expression of TSPAN13 was associated with poor survival of breast cancer patients.

Till now, reports on miR‐4732‐5p in human cancers were scarce. Omura et al have reported that five miRNAs (miR‐92b, 422a, 4732‐5p, 4758‐3p and 221) were highly expressed in gastric patients who relapsed than those who did not relapse and high expression of four miRNAs (miR‐92b, 422a, 4732‐5p and 4758‐3p) indicated poor disease‐free survival and overall survival in gastric cancer.^23^ By using a miRNA microarray, Fukumoto et al found that miR‐4732‐5p was up‐regulated in head and neck squamous cell carcinoma tissues compared with the corresponding normal tissues.^20^ Previously we found that miR‐4732‐5p was down‐regulated in nipple discharge from breast cancer patients compared to that in intraductal papilloma patients.^11^ However, the detailed function and molecular mechanism of miR‐4732‐5p in breast cancer remain largely unknown. This is the first study to reveal that miR‐4732‐5p may promote breast cancer progression at least partially by directly targeting TSPAN13. It is also noted that miR‐4732‐5p was down‐regulated in breast cancer tissues, compared with the corresponding normal tissues. We suspected that miR‐4732‐5p may act as a tumour suppressor in breast cancer initiation, however, an oncogene in tumour progression. Previous studies have demonstrated certain genes could have distinct and opposing roles in the initiation as compared with the progression of human cancers. For example, STAT3 inhibits lung cancer initiation by maintaining pulmonary homeostasis, whereas it facilitates lung cancer progression by promoting cancer cell growth.^24^ P38ɑ suppresses inflammation‐associated epithelial damage and tumourigenesis but contributes to the proliferation and survival of colon cancer cells.^25^ In breast cancer, TGFβ inhibits cell growth at early stages of carcinogenesis, but acts as an aggressive oncogene in more advanced malignant stages.^26^ We comment that the “dual role” of miR‐4732‐5p in breast cancer needs further investigation.

TSPAN13 (also known as NET‐6) belongs to the tetraspanin superfamily, which has been implicated in a range of biological processes, including differentiation, motility, proliferation and metastasis.^27^ The *TSPAN13* gene is located on chromosome 7p21.1 and encodes a 204 amino acid protein with a predicted molecular weight of 24 kDa.^28^ Geradts et al have found that expression level of TSPAN13 was lowest in breast carcinomas with aggressive characteristics 18 and TSPAN13 inhibited growth and invasion, as well as increased apoptosis of breast cancer cells in vitro and in vivo.^19^ The downstream effectors of TSPAN13 in breast cancer have been reported to include MMP‐1, MMP‐3, P53, Bax, Bak and Caspase 3.^19^ However, the upstream regulators of TSPAN13 have never been determined. Thus, our current findings provide evidence to improve characterization of the miR‐4732‐5p/TSPAN13 regulatory axis in breast cancer.

In summary, we demonstrated that miR‐4732‐5p was down‐regulated in breast cancer tissues; however, it was associated with aggressive tumour characteristics and poor prognosis. MiR‐4732‐5p promoted breast cancer cell proliferation, migration and invasion at least partially via down‐regulation of TSPAN13. Further investigations with follow‐up analysis and in vivo studies are needed to confirm the current findings, as well as the potentially dual role of miR‐4732‐5p in breast cancer initiation and progression.

## ACKNOWLEDGEMENTS

This work was supported by the Natural Science Foundation of Shandong (No. ZR2018MH029), the Shandong Key Research and Development Plan (No. 2016GSF201128), the Initial Funding for New Clinical and Practical Techniques of Qilu Hospital of Shandong University (No. 2016‐1), the Science and Technology Development Plan of Jinan (the Medical and Health Science and Technology Innovation Plan, No. 201704091), and the National Natural Science Foundation of China (No. 81402192 and 81802406). We thank Cloud‐Seq Biotech Ltd. Co. (Shanghai, China) for the mRNA‐Seq service.

## CONFLICT OF INTEREST

The authors confirm that there are no conflicts of interest.
