# Supplementary figures and images for: miR‐4732‐5p promotes breast cancer progression by targeting TSPAN13
Source: J Cell Mol Med. 2019 Jan 31;23(4):2549–57. doi: 10.1111/jcmm.14145 (PMC6433729; doi:10.1111/jcmm.14145)

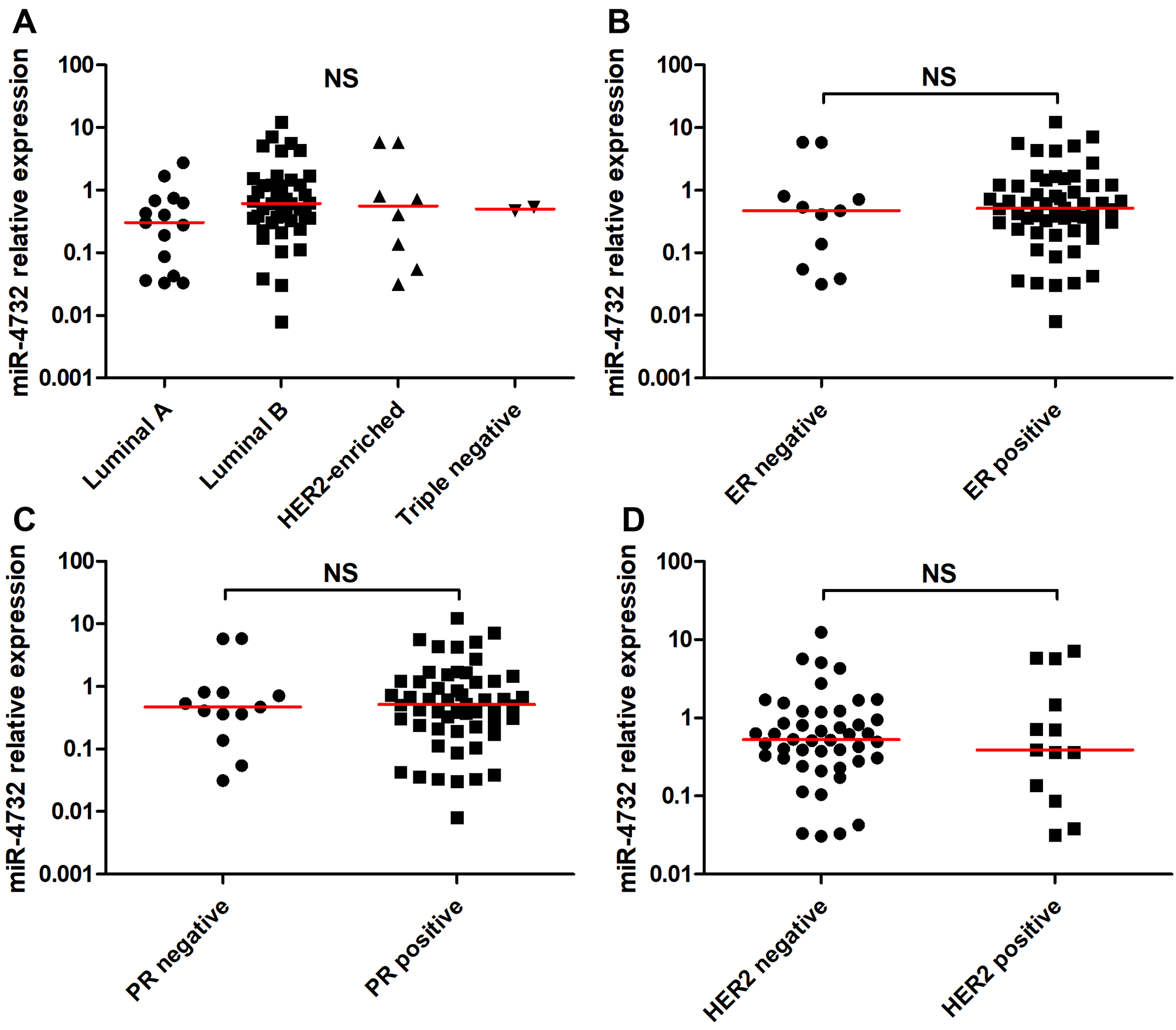

Supplement: Supplementary file 1 [file JCMM-23-2549-s001.tif]

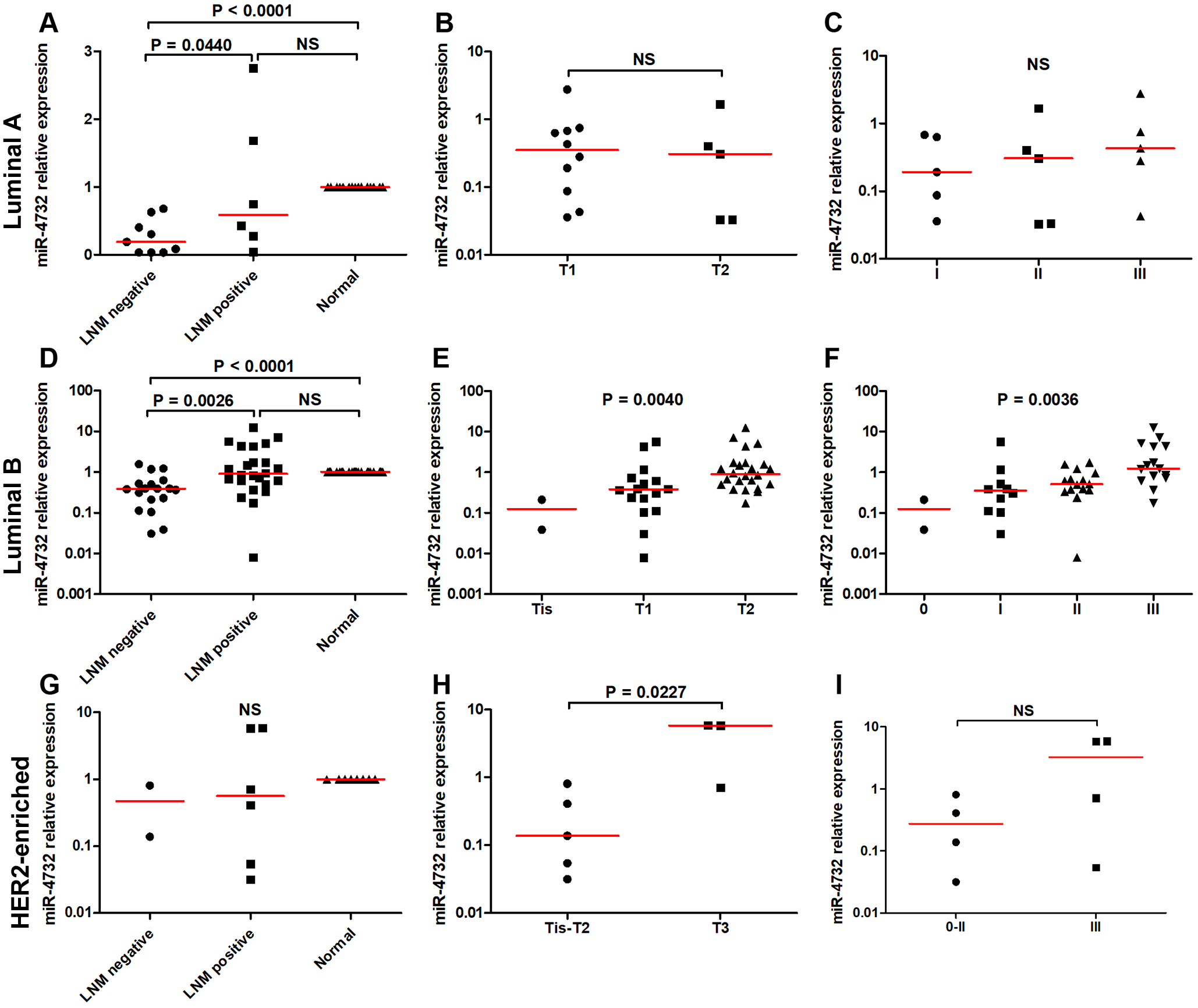

Supplement: Supplementary file 2 [file JCMM-23-2549-s002.tif]

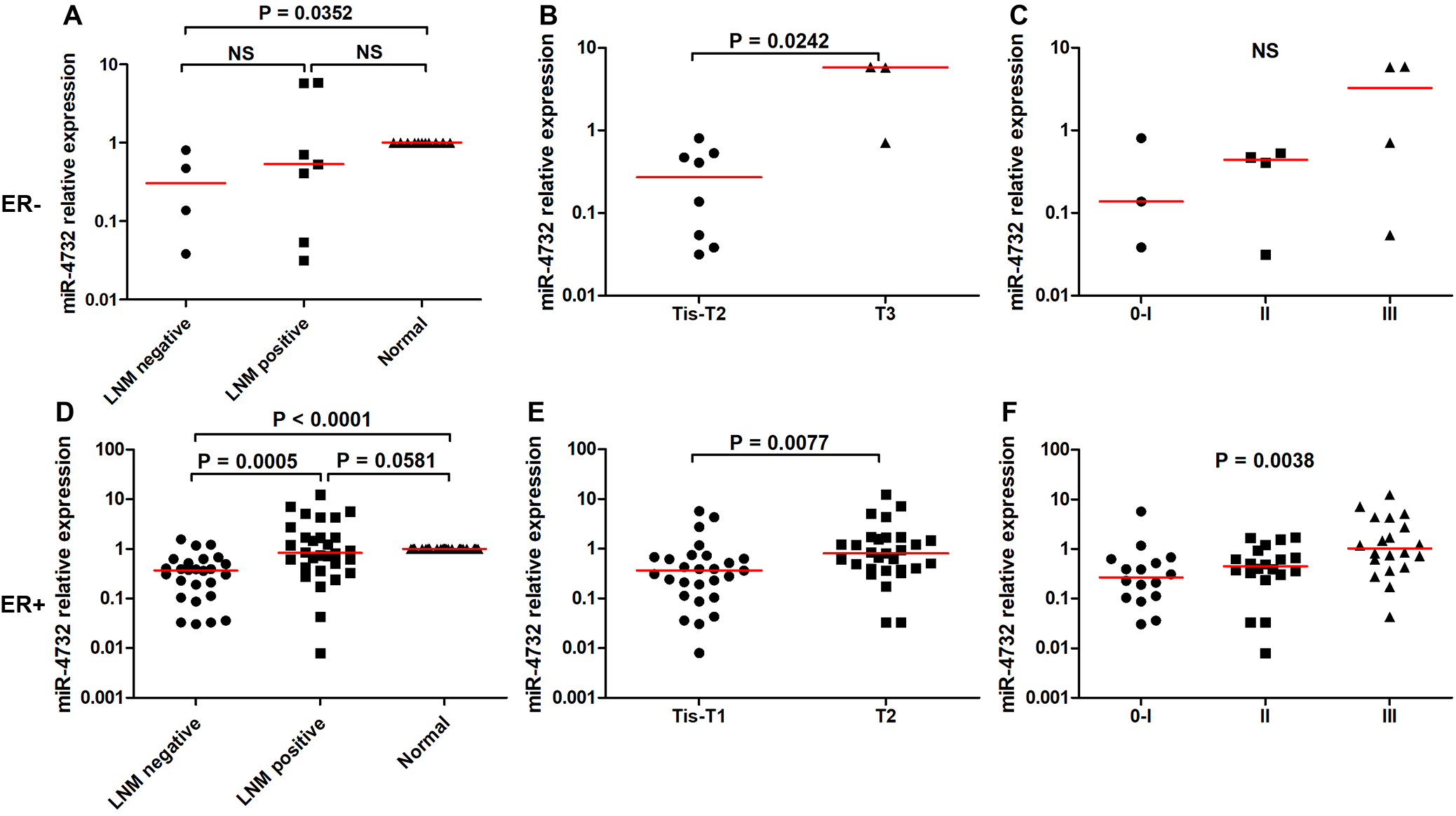

Supplement: Supplementary file 3 [file JCMM-23-2549-s003.tif]

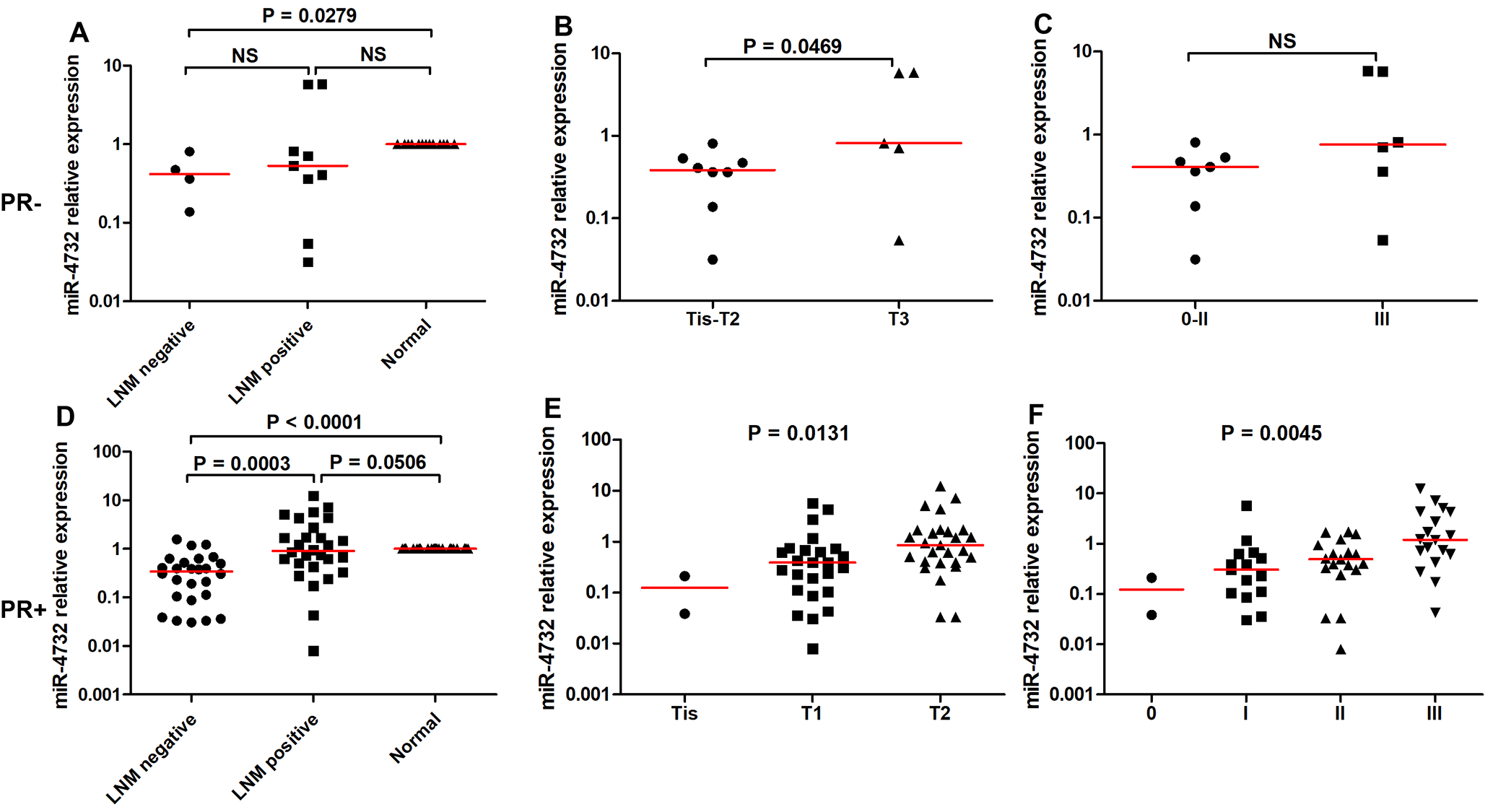

Supplement: Supplementary file 4 [file JCMM-23-2549-s004.tif]

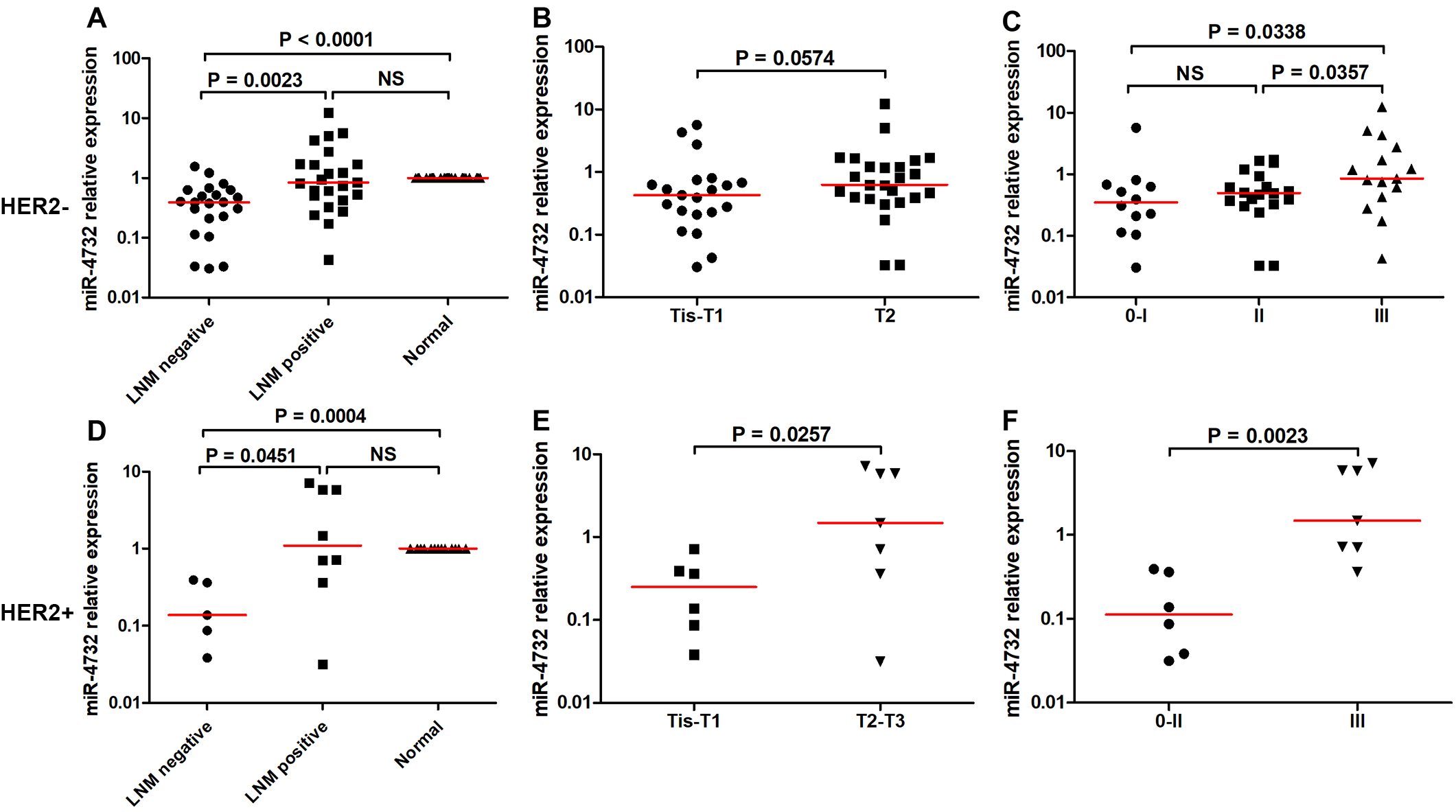

Supplement: Supplementary file 5 [file JCMM-23-2549-s005.tif]

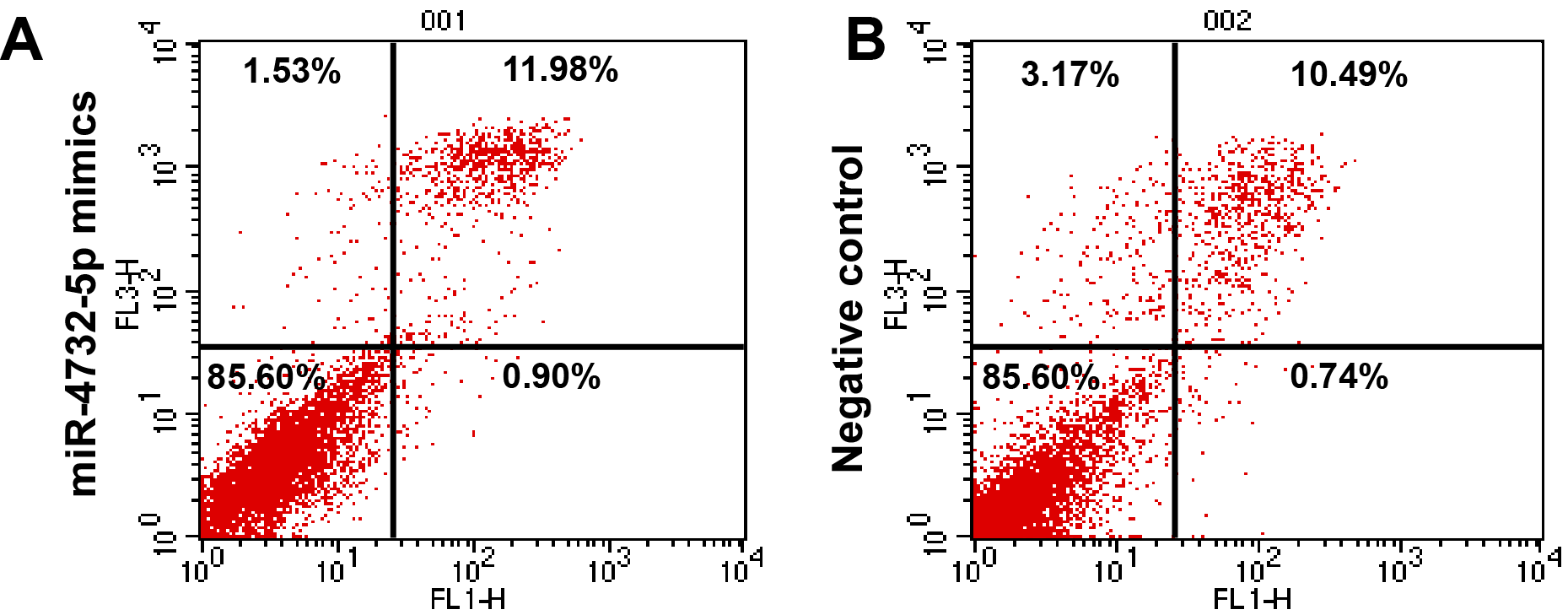

Supplement: Supplementary file 6 [file JCMM-23-2549-s006.tif]
